# Exploring Italian healthcare facilities response to COVID-19 pandemic: Lessons learned from the Italian Response to COVID-19 initiative

**DOI:** 10.3389/fpubh.2022.1016649

**Published:** 2023-01-09

**Authors:** Emanuela Parotto, Alessandro Lamberti-Castronuovo, Veronica Censi, Martina Valente, Andrea Atzori, Luca Ragazzoni

**Affiliations:** ^1^Dipartimento di Chirurgia DIDAS, Unità Operativa Complessa (UOC) Istituto Anestesia e Rianimazione, Azienda Ospedale Università, Padova, Italy; ^2^CRIMEDIM - Center for Research and Training in Disaster Medicine, Humanitarian Aid and Global Health, Università del Piemonte Orientale, Novara, Italy; ^3^Department for Sustainable Development and Ecological Transition, Università del Piemonte Orientale, Vercelli, Italy; ^4^Collegio Universitario Aspiranti Medici Missionari (CUAMM)-Doctors With Africa, Padova, Italy

**Keywords:** COVID-19 pandemic, Italian's healthcare system, health crisis, response, lessons learned, third sector, H-EDRM

## Abstract

The COVID-19 pandemic exerted an extraordinary pressure on the Italian healthcare system (Sistema Sanitario Nazionale, SSN), determining an unprecedented health crisis. In this context, a multidisciplinary non-governmental initiative called Italian Response to COVID-19 (IRC-19) was implemented from June 2020 to August 2021 to support the Italian health system through multiple activities aimed to mitigate the effects of the pandemic. The objective of this study was to shed light on the role of NGOs in supporting the SSN during the first pandemic wave by specifically exploring: (1) the main challenges experienced by Italian hospitals and out-of-hospital care facilities and (2) the nature and extent of the IRC-19 interventions specifically implemented to support healthcare facilities, to find out if and how such interventions met healthcare facilities' perceived needs at the beginning of the pandemic. We conducted a cross-sectional study using an interviewer administered 32-item questionnaire among 14 Italian healthcare facilities involved in the IRC-19 initiative. Health facilities' main challenges concerned three main areas: healthcare workers, patients, and facilities' structural changes. The IRC-19 initiative contributed to support both hospital and out-of-hospital healthcare facilities by implementing interventions for staff and patients' safety and flow management and interventions focused on the humanization of care. The support from the third sector emerged as an added value that strengthened the Italian response to the COVID-19 pandemic. This is in line with the Health—Emergency and Disaster Risk Management (H-EDRM) precepts, that call for a multisectoral and multidisciplinary collaboration for an effective disaster management.

## 1. Introduction

**Figure 1:**
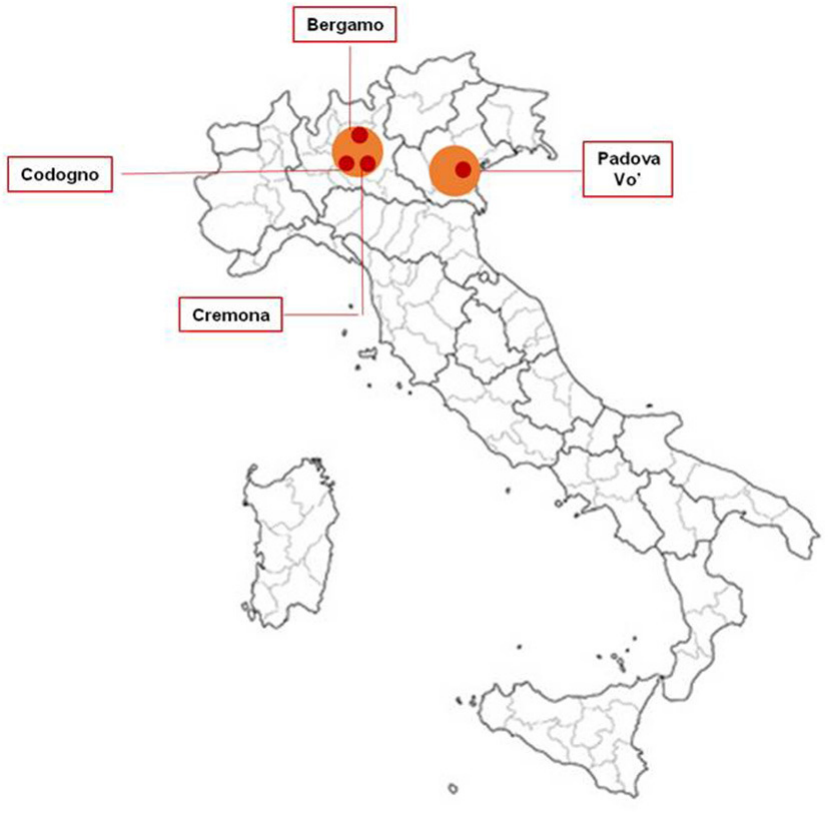
COVID-19 Italian major clusters at the beginning of the first pandemic wave.

The SARS-CoV-2 outbreak and the related COVID-19 pandemic have been the worst public health challenge in recent Italian history, placing extraordinary pressure on the country's healthcare and long-term care systems, and on the economy as a whole (1–5). COVID-19 emerged in Italy with major clusters located in northern Italy, mainly around the cities of Codogno, Bergamo and Cremona in the Lombardy region, and around the cities of Vo' and Padua in Veneto region (6) (Figure 1). Subsequently, cases spread across the country with a more sustained transmission in neighboring regions (1). Two months after the beginning of the first COVID-19 wave, the estimated excess deaths in Lombardy, the hardest hit region in the country, reached a peak of more than 23,000 deaths. This is equivalent to an excess mortality of +118% compared to the average mortality rate of the period 1st January-−30th April 2015–2019 (7). In the context of a rapidly evolving pandemic situation, the Italian Health System (Servizio Sanitario Nazionale, SSN) struggled to deal with the surge of COVID-19 patients. The most immediate challenge that the SSN faced during the first wave was the rapid saturation of hospital Intensive Care Units (ICU) capacity (8). The consequences of the spread of the virus were felt also in out-of-hospital facilities, such as nursing homes and community hospitals. Nursing homes represented particularly fragile environments in which protection strategies and training came at a later stage compared to hospitals. Personal Protective Equipment (PPE) and swab tests were in short supply for hospital staff and were even more scarce in long-term-care institutions (9). This resulted in a very rapid spread of the virus: between February, 1st and April, 14th, 2020, ~40% of deaths in the nursing homes were associated with COVID-19 (9).

**Figure 2:**
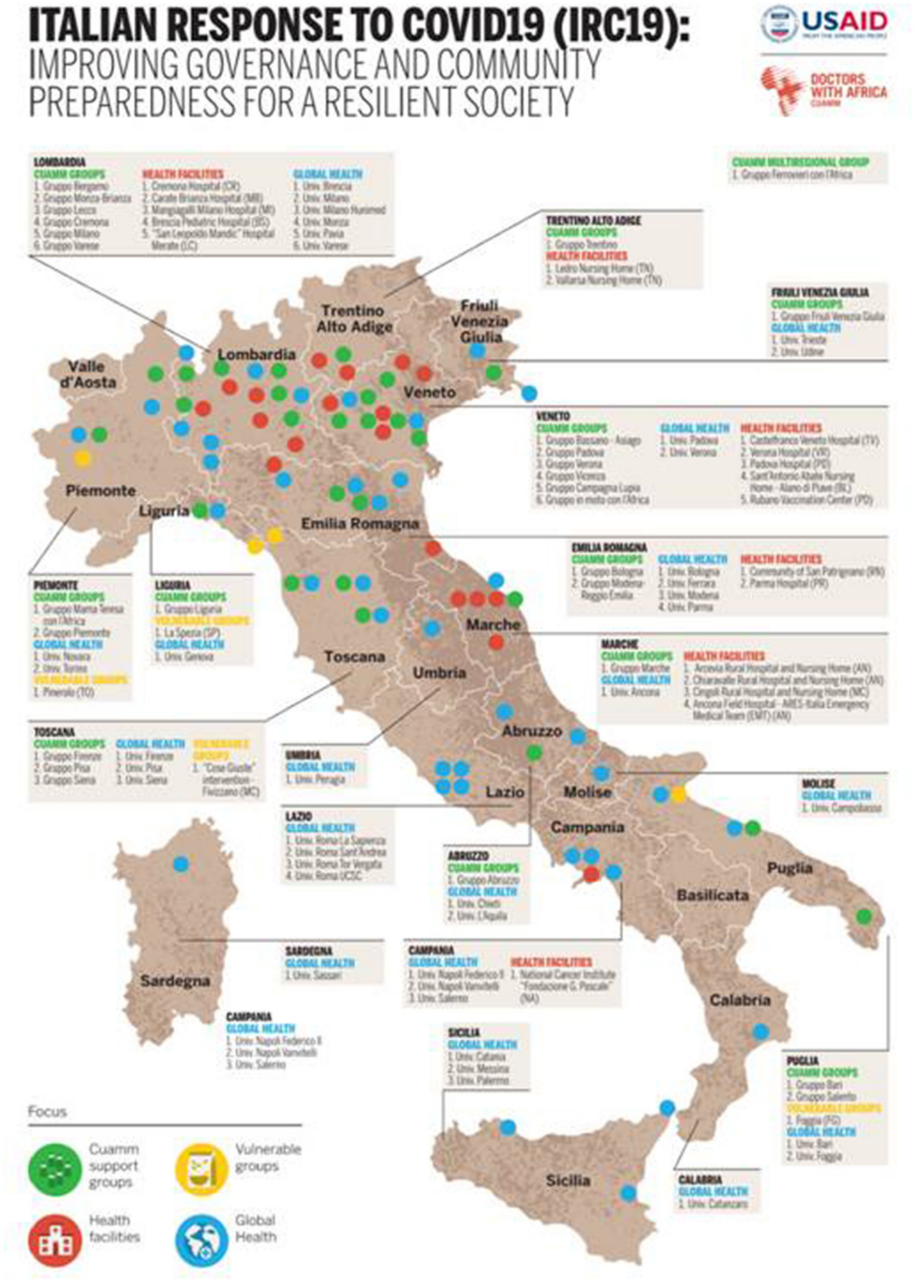
Italian Response to COVID-19 (IRC-19) initiative: map of interventions performed.

Italy developed an array of strategies to contain and mitigate the epidemic. These strategies included case-detection and contact-tracing, isolation and quarantine, physical distancing and mobility restrictions and a massive expansion of health care infrastructure and equipment (8, 10–13). Unfortunately, due to the novel aspects of the COVID-19 pandemic and to the sudden surge of patients, the SSN was unable to mount a unified response to the health crisis. Many Non-Governmental Organizations (NGOs) were mobilized to strengthen Italian healthcare facilities (14–16) and to support vulnerable populations (17, 18). The Italian NGO CUAMM—Doctors with Africa (hereinafter referred to as CUAMM) launched the Italian Response to COVID-19 (IRC-19) initiative, thanks to the support of the United States Agency for International Development (USAID) and the Center for Research and Training in Disaster Medicine, Humanitarian Aid, and Global Health (CRIMEDIM) at the University of Eastern Piedmont (Università del Piemonte Orientale, UPO), Novara, Italy (19) (Figure 2). This initiative had a duration of 14 months (June 2020–August 2021) and aimed at supporting the Italian health system through multiple activities across the territory for the prevention and mitigation of the effects of the COVID-19 pandemic (Figure 2). Within the IRC-19 initiative, four main fields of activity were defined: (1) supporting both hospital and out-of-hospital care facilities that had previously contacted the NGO asking for specific interventions to face the first pandemic wave; (2) promoting training initiatives with a focus on Global Health and Disaster Medicine topics; (3) increasing communities' awareness on pandemic-related issues; (4) providing assistance to vulnerable communities, e.g., homeless individuals (Appendix A in Supplementary material).

The objective of this study was to shed light on the role of NGOs in supporting SSN during the first pandemic wave by specifically exploring: (1) the main challenges experienced by Italian hospitals and out-of-hospital care facilities and (2) the nature and extent of the IRC-19 interventions specifically implemented to support healthcare facilities, in order to find out if and how such interventions met the healthcare facilities' perceived needs at the beginning of the pandemic.

## 2. Methods

We conducted a cross-sectional study using an interviewer-administered 32-item questionnaire (Appendix B in Supplementary material) among 14 Italian healthcare facilities from 5 different Italian regions (Lombardia, Trentino Alto-Adige, Veneto, Emilia-Romagna, Marche) involved in the IRC-19 initiative. The questionnaire was administered online from April 1st to May 31st, 2021, *via* the video conferencing tool Zoom. Two researchers conducted the interviews to collect responses to the questionnaire: one researcher actively asked the questions, and the other took notes throughout the interviews. All interviews were conducted in Italian and targeted people in managerial positions within the healthcare facilities. The average interview time was 30 min. Scientific literature was consulted (20–22) to develop the questionnaire. Such questionnaire (Appendix B in Supplementary material) predominantly relied on the World Health Organization (WHO) Emergency and Disaster Risk Management framework (H-EDRM) (23) and the United States Centers for Disease Control and Prevention (CDC) comprehensive hospital preparedness checklist for COVID-19 (24). The recommendations provided by the H-EDRM and the items selected from the CDC checklist were integrated in order to develop a guide aimed to explore the challenges experienced by the healthcare facilities related to healthcare workers, patients and structural/logistic features. Four different sections were distinguished: (a) general information about the healthcare facility; (b) basic characteristics of the facility; (c) main challenges experienced by the facility at the beginning of the first COVID-19 pandemic wave; (d) interventions implemented through the IRC-19 initiative. Data was collected in each healthcare facility in the period from June 1st, 2020, to April 1st, 2021. Each section included multiple choice questions that were integrated with additional comments provided by participants. Count, frequency, percentage, and mean/median scores were used to report descriptive statistics. The limited sample size and heterogeneity among healthcare centers did not allow to assess any statistical inference. Qualitative thematic analysis was used to analyze the open-ended answers provided by participants to supplement multiple choice questions (25). The qualitative analysis was performed following the Consolidated Criteria for Reporting Qualitative Research (COREQ) (26). Both descriptive statistical and qualitative results were reported as integrated with each other.

Stakeholders were contacted *via* email and information on the study objective, methodology and ethical implications was reported in the email. Written informed consent was obtained by all participants. The study was conducted according to the Declaration of Helsinki guidelines and approved by the Ethics Committee of the A.O.U. “Maggiore della Carità” in Novara (Study Number 015.059).

## 3. Results

### 3.1. Characteristics of the healthcare facilities

The 14 healthcare facilities that took part in this study belonged to the following categories: nursing homes (*N* = 5); community hospital (*N* = 1); community for people with addiction problems (*N* = 1); tertiary hospital wards (*N* = 5); field hospital (*N* = 1). Community hospitals are intended as short-term hospitalization facilities aimed to support patients who require low clinical intensity health interventions and who need continuous nursing health care/surveillance that is not available at home. The number of beds for each healthcare facility ranged from 38 to 1,220. The number of hospital admissions per year, reported per hospital ward, ranged from 3,600 to 40,000 (Table 1). The interviewed people were nurses (*N* = 7) and medical doctors (*N* = 7). All of them had managerial roles.

**Table 1:** Healthcare facilities involved in the study.

**Region**	**Province**	**Health facilities involved**	**Patients admission capacity**	
			**Number of beds**	**Number of hospital admissions/year (2019)**
Lombardia	Cremona, CR	Emergency department	-	40,000
	Monza—Brianza, MB	Emergency department	-	40,000
	Milano, MI	Obstetric ward	114	5,300
	Brescia, BS	Pediatric emergency department	-	3,600
	Lecco, LC	Pediatric ward	18	-
Trentino	Trento, TN	Nursing home	38	-
	Trento, TN	Nursing home	60	-
Veneto	Belluno, BL	Nursing home	63	-
Emilia Romagna	Ravenna, RA	Community for people with addiction problems	1,200	-
	Parma, PR	Pediatric emergency department	-	20,000
Marche	Macerata, MC	Community hospital	40	-
	Ancona, AN	Nursing home	54	-
	Ancona, AN	Nursing home	40	-
	Ancona, AN	Field hospital	150	-

### 3.2. Main challenges experienced by healthcare facilities

**Figure 3:**
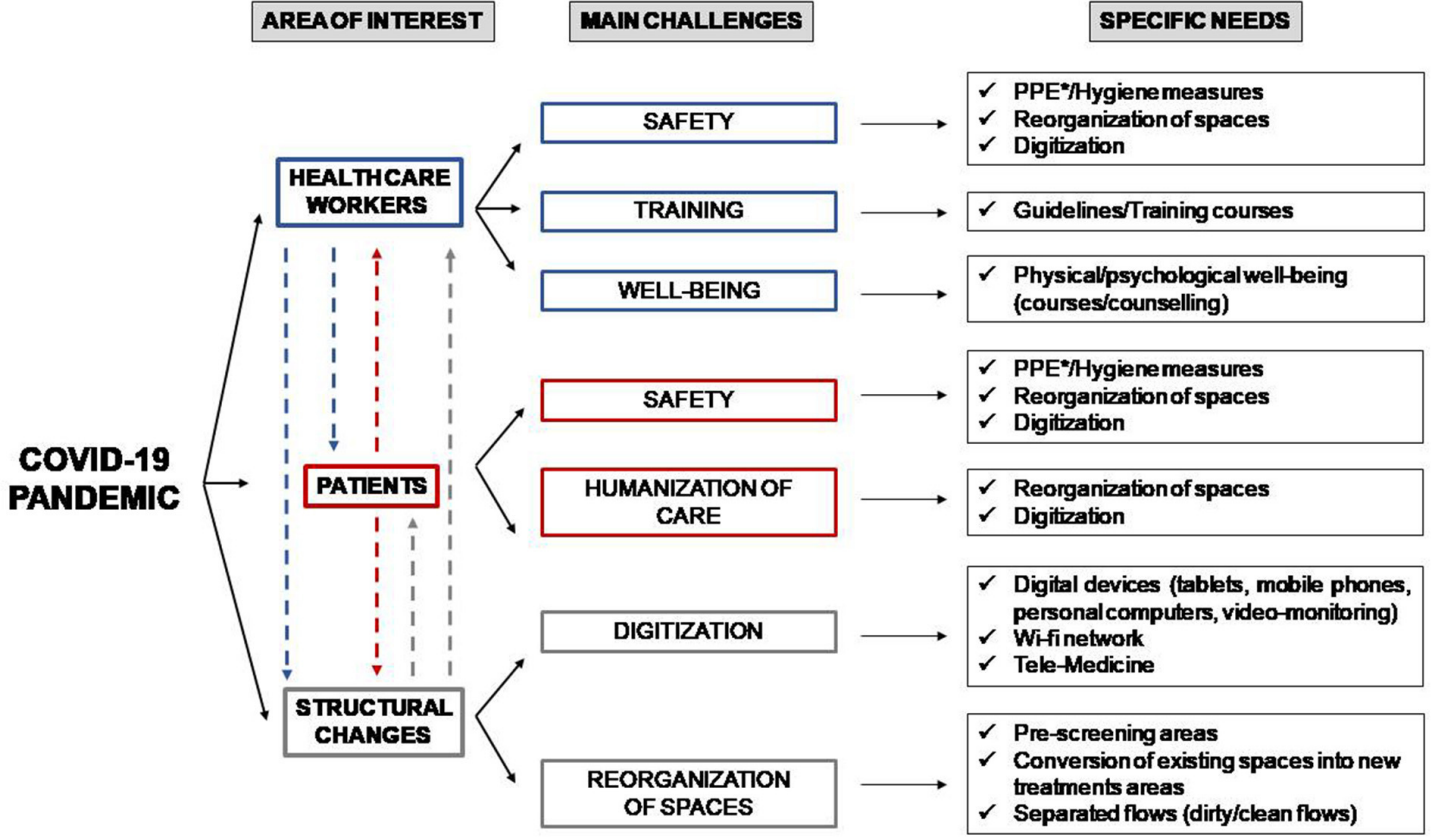
Main challenges experienced by both hospitals and out-of-hospital care facilities during the first pandemic wave. The three main areas of interest (healthcare workers, patients and structural changes) showed interdependence among each other. The interdependence is illustrated in the figure with dashed lines. *PPE, Personal Protective Equipment.

The challenges that Italian healthcare facilities faced during the first COVID-19 wave can be categorized in three main areas: (1) maintaining and supporting healthcare workforce (safety, training, and wellbeing), (2) providing care to patients, and (3) improving the infrastructure of the healthcare facilities (digitization and structural changes) (Table 2; Figure 3).

**Table 2:** Main challenges reported from different Italian healthcare facilities during the first COVID-19 pandemic wave (PPE, Personal Protective Equipment).

**Areas of interest**	**Main challenges experienced**	**Specific needs**	**Number of health facilities (*N*_total_ = 14)**	**Percentage (%)**
Healthcare workers	Safety	PPE supply	9	64
		Hygiene measures	10	71
		Reorganization of spaces	12	86
	Training	Training courses	14	100
	Wellbeing	Psychological wellbeing	12	86
Patients	Safety	Reorganization of spaces	12	86
	Humanization of care	Digitization and reorganization	12	86
Health care facilities	Structural changes	Reorganization of spaces	12	86
	Digitization	Wi-Fi implementation	7	50
		Personal computer purchase	6	43
		Tablets purchase	8	57
		Smartphones purchase	2	14

The challenges that the sudden surge of COVID-19 patients determined in each of the above mentioned sectors (healthcare workforce, patients, and healthcare facilities' infrastructure) are interdependent among each other. As an example, the surge in COVID-19 patients created challenges with HCWs' safety, training, and wellbeing (Figure 3). It also led to specific structural changes in the health facilities to expand existing treatment areas. The subsequent reorganization of spaces within healthcare facilities allowed to implement infection prevention and control measures and a safe management of patients, consequently leading to a redistribution of HCWs themselves within the healthcare facilities.

Most of the respondents (93%) considered safety of HCWs a primary concern. Issues in PPE availability and the lack of hand wash points were reported by 64% and 71% of the facilities, respectively: “*At the beginning of the first pandemic wave we didn't have facial masks, not even surgical ones” (Nursing home, Trentino Alto-Adige); “At the beginning of the first pandemic wave we experienced a shortage of PPE. When we had the first positive patient in our facility, we didn't have adequate PPE to protect ourselves and other patients. Subsequently, the situation improved, but at first the supply was sufficient only for COVID-19 wards and not for all health professionals and patients” (Community hospital, Marche)*. Adequate training of HCWs concerning the proper use of PPE and the managing of COVID-19 patients was considered a priority by all respondents in order to ensure safety of the health professionals: “*After receiving the proper training about the virus, we were able to overcome our fears” (Nursing home, Veneto)*. Moreover, 86% of the participants underlined the importance of ensuring the separation between dirty and clean areas. Alongside physical safety, a vast majority of respondents (86%) reported the need to manage severe psychological stress and to protect health workers' wellbeing as a major priority. The psychological stress was due to the spread of an unknown and severe disease with a high number of victims and to the need of working in unfamiliar environments with new protocols: “*We were worn out and devastated. We have had a lot of deaths among our patients” (Nursing home, Trentino Alto –Adige); “The beginning of the pandemic felt almost like being swept away by an avalanche...At the beginning of the pandemic our pediatric unit was transformed into an adult COVID-19 unit. This change was extremely stressful for our healthcare workers, not used to managing adult patients” (Pediatric Hospital ward, Lombardia); “We were overwhelmed by the emergency and the massive influx of patients…We cried in the wards, in the offices…but we didn't have the time to organize psychological support for our workers” (Hospital, Lombardia)*. Protection of patients' safety emerged as a critical challenge in 86% of the health facilities considered. Many participants considered the reorganization of pre-existing spaces through the purchase of specific furnishings (62%) and the installation of automated doors (38%) as fundamental. This was done in order to ensure the separation between dirty and clean areas: “*It was very difficult to guarantee the different dirty/clean paths due to the structural characteristics of our building” (Nursing home, Trentino Alto –Adige)*; “*In our facility we had tight spaces … it was difficult to guarantee community life in safe conditions to our guests” (Nursing home, Veneto)*. In addition, many respondents (69%) underlined the need to develop new structures aimed to guarantee an adequate treatment area both for COVID-19 and non-COVID-19 patients. In order to ensure the best possible treatment to patients, many respondents (86%) considered providing humanized care despite the strict isolation measures imposed by the emergency as necessary (“*our structure is built to foster community life”, Nursing home, Trentino Alto–Adige)*. The quick implementation of digital services was considered a major need at the beginning of the first pandemic wave. Many participants regarded the implementation of the Wi-Fi system (50%) and the purchase of digital devices (personal computers, 43%; tablets, 57% and mobile phones, 14%) as fundamental. These initiatives served to improve working conditions and the humanization of care.

### 3.3. IRC-19 interventions

The interventions implemented within the IRC-19 initiative can be grouped in three main sectors: (1) interventions for patients and staff safety; (2) for the positive/negative flow management of patients/HCWs; (3) for the humanization of care (Table 3).

**Table 3:** IRC-19 interventions group according to five Italian regions involved in the study (PPE, Personal Protective Equipment).

**Region**	**Safety (health care workers/patients)**	**Flow management**	**Humanization of care**
Lombardia	• ***Safety*** - Wash points for hands hygiene - Changing rooms for health care workers - Waiting room - External canopy - Workroom with a personal computer station - Radiolucent stretchers, mattresses, washable cushions - Movable dividers to ensure privacy for patients - Maternal and fetal multi-parametric monitors - Video surveillance system in patients' room - Breast pumps - Neonatal heating mats	• ***Structural changes*** - Refurbishment and integration of horizontal and vertical external signs for the access-exit routes - Movable dividers - Automatic doors	• ***Digitization*** - Tablets - Smartphones
Trentino Alto—Adige	• ***Safety*** - Wooden prefabricated where health care workers can wear uniforms and PPE • ***Training*** - JUST IN TIME course • ***Wellbeing*** - FIT4CARE course	• ***Structural changes*** - Automated doors	• ***Structural changes*** - Wooden prefabricated house to schedule visits with relatives - Glazed windows areas to allow interactions between residents and to schedule visits with relatives
Veneto			• ***Structural changes*** - Gazebo to guarantee outdoor activities for residents and meeting with relatives
Emilia—Romagna	• ***Safety*** - Personal computers, tablets, speaker phone, wide-angle webcam, wireless keyboard/mouse to implement information system technology • ***Training*** - JUST IN TIME course • ***Wellbeing*** - FIT4CARE course	• ***Structural changes*** - Plasterboard - Doors - Tents	• ***Digitization*** - Multi-media units to allow telemedicine and virtual meetings between patients and their relatives - Wi-Fi system
Marche	• ***Safety*** - Wash-points for hands hygiene - PPE storage cabinet - Lockers for health care workers with double compartments (clean/dirty) - Wi-Fi to allow data transmission and video calls - Personal computers, tablets, printers, to implement information system technology - Sanitizable chairs - Furniture for the storage of contaminated waste • ***Training*** - JUST IN TIME course • ***Wellbeing*** - FIT4CARE course	• ***Structural changes*** - Automated doors, movable dividers - Cabinet for storage of sanitizing materials - Microwave oven to allow the heating of infusion liquids and drugs - Freezers that allow to eliminate pre-packaged ice packs that occupy large volumes of space - Thermometers that allow to monitor the temperature for the storage of drugs - Furnishing accessories (chairs, tables)	• ***Structural changes*** - Furnished room to allow communication between healthcare workers and relatives • ***Digitization*** - Tablets to allow video calls - Smart TV for living room and for patients isolated in their rooms • ***Others*** - Sanitizable toys for occupational therapy for patients suffering from cognitive impairment

IRC-19 interventions for patients and staff safety aimed at creating a safer environment for both HCWs and patients, reducing the risk of COVID-19 infection while ensuring the performance of the services and activities (“*Feeling taken into consideration made us stronger and more confident in dealing with the pandemic”, Nursing home, Trentino Alto-Adige)*. The interventions performed included assembling temporary infrastructures (29%), purchasing new furniture (43%) and new lockers for HCWs (21%), renovating existing structures with the creation of new spaces (57%), installing new automated doors (21%) and hand wash-points (43%), creating warehouses for the storage of PPE (7%). The IRC-19 initiative contributed to ensuring occupational health and safety strengthening HCWs' training with the realization of two free modular training packages called JUST IN TIME and FIT4CARE, performed in 64% of the health facilities enrolled in this study: “*FIT4CARE course was much appreciated…concerning the issue of health professional well-being we didn't receive support from our hospital” (Pediatric ward, Emilia—Romagna)*.

The IRC-19 interventions for the positive/negative flow management of patients/HCWs aimed at managing the flows of patients, residents, visitors and HCWs entering and leaving the facilities and preventing the spread and contamination of the virus. This was possible thanks to the improvement and re-organization of the entry-exit routes of the facilities (21%), and the purchase of specific furnishings such as movable dividers to guarantee patients' privacy or to perform medical visits in safety (36%).

Many IRC-19 interventions were aimed at ensuring humanized care despite the isolation measures. The interventions performed ranged from the implementation of the Wi-Fi network (50%) to the purchase of mobile phones and tablets (64%): “*Thanks to the IRC-19 initiative we implemented a tele-surveillance system for COVID-19 patients during labor and after delivery” (Obstetric ward, Lombardia)*; “*We received tablets that allowed our COVID-19 patients to communicate with their relatives and to see their newborns in case they were admitted to Neonatal Intensive Care Unit after the delivery” (Obstetric ward, Lombardia)*. Moreover, prefabricated buildings (36%) were constructed, where family visits were allowed: “*The family house given by this project represents an important additional value for our institute and our community. It has gathered admiration and positive comments among public opinion and in the media. It gave us the opportunity to react and to leave no one behind” (Nursing home, Trentino Alto-Adige); “Thanks to this project we had the possibility to re-organize our spaces for meetings and social interaction for our residents...Thanks to this project we will be able to open our institute to the community” (Nursing home, Veneto)*.

Notably, these interventions promoted the use of telemedicine both for patients and HCWs: “*Thanks to these interventions we have managed to maintain the psychological well-being of our guests. We have managed to carry out the activities that are essential for our community. During the pandemic period we have not observed an increase in dropout rates among our guests” (Community for people with addiction problems, Emilia Romagna)*.

## 4. Discussion

The main needs experienced by different Italian healthcare facilities concerned three main areas: HCWs (safety, training, and wellbeing), patients (safety and humanization of care) and structural changes (reorganization of spaces and digitization). Specific strategies were implemented to meet such needs. Despite the different specificities, it should be noticed that the three main groups mutually interacted (Figure 3), suggesting that they have to be considered all together in planning successful pandemic responses. With regard to the IRC-19 interventions, it is possible to identify three main target areas: interventions targeting staff and patients' safety, interventions targeting patients/health professionals flow management and interventions aimed to ensure humanization of care (Table 3). The entirety of the IRC-19 interventions supported the healthcare facilities involved in this study by promptly addressing the main needs experienced by the healthcare facilities during the first pandemic wave.

The shortage and improper use of PPE, that was mainly seen in out-of-hospital facilities, represented one of the major challenges encountered. This confirms the results of previous studies targeting nursing homes that highlighted not only the shortage of PPE during the first pandemic wave but also the late implementation of response strategies at this level compared to hospitals (27). The need to reshape health services delivery by improving primary and community care provision was already suggested in recent publications as a fundamental strategy to face health pandemic challenges and render communities more resilient (28). Strengthening primary and community care services might contribute to reducing the surge of avoidable hospitalizations for Ambulatory Care Sensitive conditions (ACSCs) that persist in the long term after disasters, as it was shown in a recent literature review (29). Furthermore, the respondents considered adequate training of HCWs as fundamental to guarantee safety and protection and to face the impact of the pandemic, reflecting findings reported in previous studies (30). Concerning HCWs' wellbeing, a relevant challenge identified in this study was the significant psychological stress to which the staff was exposed during the first pandemic wave. Scientific literature has previously documented the negative impact on HCWs' psychological health during the pandemic (24, 25). This is mainly attributable to the increased workload together with the shortage of adequate PPE and the absence of an evidence-based treatment (24, 25). The lack of adequate training (30, 31), the fear of contagion (32) and the management of quickly deteriorating patients (32–34) are additional factors mentioned in the literature.

The COVID-19 pandemic has altered the way patients and families endure illness and death and has emphasized the importance of being culturally prepared to face suffering and death to such a large extent (35–38). In this study, the lack of a “humanized care” was mainly felt in nursing homes and community hospitals, where patients' relational aspects with the community and the outside world are of paramount importance. Similarly, obstetric and pediatric units underlined the need to protect relationships between mothers and children despite the restriction measures.

With regard to the health facilities' structural changes described in this study, several measures were reported as fundamental to guarantee adequate infection prevention and control: (1) the ability for hospitals to retrofit or reallocate parts of their facilities in order to ensure separate emergency entrances for contagious patients, (2) having patients' pre-screening and treatment areas; (3) having separation between patients, visitors, and staff, based on their level of contagion. These are all measures that need to be considered when defining health facilities' preparedness strategies (39).

In addition to structural changes, the implementation of digitization was reported as a relevant measure both to facilitate relationships between HCWs/patients/caregivers and to allow remote medical consultations. The adoption of telemedicine emerged as a global theme during the COVID-19 pandemic (35–37). In this regard, the Italian Ministry of Health, together with the Ministry for Technology Innovation and Digitization and the WHO, launched an open call to implement telemedicine and monitoring system technologies (40) in the health facilities. However, many Italian hospitals lack adequate infrastructure for effective telemedicine platforms, due to supply-chain breakdown and insufficient internet capabilities (41). The IRC-19 initiative contributed to address these needs providing support with the implementation of digital technologies.

The first IRC-19 intervention area was aimed to guarantee staff and patients safety through structural changes and training strategies. Structural changes included the creation of specific prefabricated buildings, the purchase of specific furniture and devices (automated doors, lockers, wash points for hand hygiene, warehouse for PPE storage) and the renovation of existing structures with the creation of new spaces and the installation of temporary infrastructures. The measures taken were considered as essential by most of the participants in order to establish an adequate Infection Prevention and Control (IPC) system within the healthcare facilities affected by the pandemic crisis.

Training strategies were developed as online teaching learning sessions (OTL) and included the JUST IN TIME and the FIT4CARE courses. The JUST IN TIME course focused on disasters and COVID-19 management principles and offered take-home messages to support healthcare workers struggling with the lack of knowledge and the absence of evidence-based treatments. The FIT4CARE course was aimed to improve healthcare professionals' wellbeing offering feasible and easy-to-apply tools provided by experts in Nutrition, Fitness training and Psychology. It facilitated healthcare workforce in facing the intense physical and mental fatigue experienced during the first pandemic wave.

The second IRC-19 intervention area focused on the appropriate flow-management within the healthcare facilities. It included the purchase of specific furniture (movable dividers and automated doors), the installation of new buildings (gazebos and tents) and the delineation of different clean/dirty pathways. Reorganizing spaces efficiently was considered as a fundamental intervention in most of the healthcare facilities involved in this project due to the fact that it helped to create a safer environment reducing the risk of the contagion and the spread of the virus.

The third IRC-19 intervention concerned the need to ensure humanized care with a patient-centered approach despite social distancing and restrictive safety measures. The possibility to guarantee an appropriate relationship between patients and HCWs and between patients and their families was an important need experienced both in hospital wards and in community hospitals/nursing homes. In hospitals, interventions to guarantee the humanization of care were mainly requested in maternal and obstetric units. The installation of a Wi-Fi monitoring and of a video/audio system for maternal and fetal surveillance in each patient room allowed health professionals to constantly interact with patients during labor and after delivery, reducing movement across different areas. The purchase of tablets ensured the communication in the post-partum period between mothers and their newborns or relatives in case of women with prolonged hospital stay or newborns admitted in Neonatal Intensive Care Unit. The purchase of breast pumps and heating mats were aimed at protecting and maintaining the relationship between mothers and newborns in the COVID-19 maternal unit. In nursing homes maintaining human relations was considered fundamental for the protection of patients' health. The installation of new buildings (e.g., wooden houses and a gazebo with lighting and heating systems) allowed to schedule visits of family members in accordance with safety procedures. Moreover, the implementation of digitization systems (Wi-Fi, personal computers, webcam) helped guests to maintain some contact with their relatives and allowed telemedicine consultations.

The digitization measures adopted thanks to the support of the IRC-19 initiative played an important role in assisting healthcare facilities to face multiple pandemic challenges. The implementation of digital technologies was perceived as fundamental to ensure the continuity of care through remote medical consultations and to support the relationships between patients and family members in spite of the containment measures. Moreover, the possibility to provide medical consultations remotely reduced the risk of contagion both for healthcare professionals and patients contributing to creating a safer environment and to reducing the fear of contagion.

In conclusion, the IRC-19 initiative supported Italian health care facilities in facing the main challenges encountered during the first pandemic wave, stepping in to fill some unmet needs when the SSN was overwhelmed. Recent studies showed that NGOs globally played a significant role when national governments alone couldn't manage to fulfill the needs of the population (42–44), but scientific publications concerning this topic were missing at the time of writing this manuscript. The findings of this study confirmed the results of previous publications that have emphasized the importance of NGOs' role in supporting countries when emergencies and disasters occur (45, 46). The solid working experience with vulnerable and marginalized people in low resource settings, the closer connection with the communities and the adoption of more flexible bureaucratic processes were identified among the main factors that allow these organizations to respond more quickly to crises (43).

These reflections are closely related to the main foundations reported by the H-EDRM framework (23). The effective management of the challenges that the COVID-19 pandemic posed to the Italian health system required multisectoral and multidisciplinary collaboration to be solved. Recent findings shown that the interconnection of different sectors (e.g., PHC, hospital and third sector) with a decentralized distribution of services to primary and community care was key for overcoming challenges posed by the COVID-19 pandemic (30). Within the context of multisectoral collaboration, strong relationships between healthcare facilities and the third sector represented an irreplaceable strategy to face the pandemic disaster during the first wave.

### 4.1. Limitations

The main limitations concerned the small sample size and the heterogeneity of the healthcare facilities involved. The small sample size didn't allow to assess any statistical inference concerning quantitative data. Nevertheless, the number of participants interviewed followed the principle of qualitative data saturation in accordance to the Consolidated Criteria for Reporting Qualitative Research (COREQ) (26). The heterogeneity of the sample encompassed both the different patients' admission capacity and the different typology of healthcare facilities involved. Although the absence of an homogeneous population entailed some important limitations, it offered a broader set of perspectives to evaluate the impact of COVID-19 pandemic on the Italian health system.

In addition, we considered health care facilities supported by the same non-governmental project. Hence, other strategies in support of the SSN (i.e., other non-governmental organizations, private sector, and private-public partnerships) were not considered. Further studies are needed to expand the sample and to prove the involvement of the third sector in the health management of disasters.

## 5. Conclusions

The COVID-19 pandemic has exerted extraordinary pressure on the entire SSN, both on hospitals and out-of-hospital healthcare facilities. The crisis of the first pandemic wave made it difficult for the SSN to homogeneously guarantee an immediate and effective support to all the challenges experienced by the different Italian health care facilities. In this complex context, the IRC-19 initiative represented an instrument to fill this gap, allowing to support and strengthen both hospitals in the frontline against the virus as well as out-of-hospital healthcare facilities that were nevertheless severely hit. The support from the third sector emerged as an added value that strengthened the Italian response to the COVID-19 pandemic disaster.

## Data availability statement

The raw data supporting the conclusions of this article will be made available by the authors, without undue reservation.

## Author contributions

EP designed the study and drafted the manuscript. EP and VC conducted the data collection. AL-C, MV, VC, AA, and LR completed and revised the drafted manuscript. All authors have approved the final version of the manuscript for submission.
