# Supplementary material for: Exploring Italian healthcare facilities response to COVID-19 pandemic: Lessons learned from the Italian Response to COVID-19 initiative
Source: Front Public Health. 2023 Jan 9;10:1016649. doi: 10.3389/fpubh.2022.1016649 (PMC9870543; doi:10.3389/fpubh.2022.1016649)
Supplement: Supplementary file 1 [file Table_1.docx]

**SUPPLEMENTARY MATERIALS**

**Appendix A. Italian Response to COVID-19 initiative: main intervention areas.**

| **ITALIAN RESPONSE TO COVID-19 (IRC-19) INITIATIVE** |
| --- |
| The Italian Response to COVID-19 (IRC-19) initiative aimed to restart and support multiple activities on Italian territory for the prevention and mitigation of the effects of the COVID-19 pandemic. The project promoted the support of health care facilities and the protection of specific vulnerable groups during the COVID-19 pandemic crisis. IRC-19 interventions focused on four different main interventional areas:  **1. Improvement of healthcare facilities**  Interventions aimed to support hospitals and out-of-hospital health services in order to improve infrastructures and hygiene practices, patient care and health professionals safety. The time required for the completion of these interventions was 3-8 months and the budget ranged between 4,000 and 35,000 euro. The interventions focused on: a) rehabilitation of spaces, with specific planning for each reality according to the specific needs and in collaboration with regional technicians and managers of the different facilities; b) training of HCWs with the JUST IN TIME course; c) strengthening of HCWs’ well being with the FIT4CARE course.  *IRC-19 Training courses*  JUST IN TIME course: free modular training package supported by CRIMEDIM (Center for Research and Training in Disaster Medicine, Humanitarian Aid and Global Health) and focused on disasters and health emergencies management in healthcare facilities. It included two different phases. The first phase was a distance learning phase (FAD) on a Moodle platform consisting of six modules with presentation of video lessons realized by different instructors. The second phase was a live online session divided into three different sessions delivered via the ZOOM platform conducted by different instructors. The purpose of the second phase was to discuss in depth all the topics addressed by the first phase through frontal lessons and interactive sessions.  FIT4CARE course: free digital training package developed in collaboration with experts in Psychology, Nutrition and Fitness and sponsored by the Presidency of the Council of Ministers. The aim of the course was to provide simple and easy-to-apply tools to improve the state of mental and physical health of HCWs. The course consisted of six video sessions dedicated to the themes of Physical Preparation, Stretching, Nutrition, Psychology and Breathing. Upon the completion of the video lessons, there was a free online session of about two and a half hours with a trainer for an in-depth study of the topics previously addressed.  **2. Improvement of community awareness**  Interventions performed through CUAMM’s network support groups (27 groups in 11 Italian regions) aimed to expand community awareness and resilience in the context of the COVID-19 pandemic.  **3. Assistance to vulnerable groups**  Intervention aimed to support two realities of vulnerability on the Italian territory: a) provision of assistance to seasonal farm laborers in the areas of Borgo Mezzanone, Casa Sankara and Arena, in the Puglia region (Italy); b) provision of assistance to homeless people and to people that had experienced financial difficulties due to the pandemic crisis, in the Liguria region (Italy).  **4. Global Health education**  Interventions aimed to promote Global Health education among health professionals and performed through the involvement of the Italian Secretariat of Medical Students (Segretariato Italiano degli Studenti di Medicina, SISM), the National Association of Medical Residents (FederSpecializzandi) and the Italian Secretariat of Young Medical doctors (Segretariato Italiano Giovani Medici, SIGM). |

**Appendix B. Interviewer-administered questionnaire used for data collection.**

**Section 1.** *General data.* This section of the questionnaire focused on general data about the healthcare facilities and the respondents.

| **FACILITY CENSUS QUESTIONNAIRE** | | |
| --- | --- | --- |
|  | **Question** | **Answer** |
| 1 | Date (dd/month/year) |  |
| 2 | Facility Region/City |  |
| 3 | Facility name |  |
| 4 | Facility address |  |
| 5 | Facility telephone, e-mail and fax numbers | Telephone:  e-mail:  fax: |
| 6 | Interviewer name (last, first) |  |
| 7 | Respondent name (last, first) |  |
| 8 | Respondent job title |  |

**Section 2.** *General characteristics.* This section of the questionnaire focused on the basic characteristics of the facility including different subsections for patients and healthcare workers (HCWs).

| **GENERAL CHARACTERISTIC** | | | |
| --- | --- | --- | --- |
|  | **Question** | **Answer/Notes** | |
| 1 | Type of facility | - Hospital:  1. first level * 2. second level**  - Nursing Home - Community Hospital ^§^ - Other (*specify*) |  |
| 2 | Unit of interest | - Emergency Department - Hospital wards - Hospitalisation Units < 30 days *(Lungodegenza)* - Hospitalisation Units > 30 days days *(RSA)* |  |
| 3 | Patients | Numbers of daily access |  |
|  |  | Numbers of beds |  |
|  |  | Patients access routes to the facility | - dedicated entrance *(specify)* - shared entrance (HCWs, patients, caregivers, administrative employees) - Other *(specify)*   *______________*  (w |
| 4 | Healthcare  workers | Numbers of health workers |  |
|  |  | Professional category of interest | - Auxiliary nurse - Nurse - Physician - Administrative employee |
| *First level hospital: hospital with a catchment area of 600.000-1.200.000 inhabitants  **Second level hospital: hospital with a catchment area: 300.000-600.000 inhabitants  ^§^ Community hospitals: healthcare facilities intended for short-term hospitalizations for patients in need of low clinical intensity health interventions | | | |

**Section 3.** *Interventions needed.* This section aimed at identifying the main interventions needed to implement the response to COVID-19.

| **INTERVENTIONS NEEDED** | | |
| --- | --- | --- |
| **Group** | **Issue** | **Needs** |
| Healthcare workers | 1. Safety | - Personal protective equipment - Wash points - Different pathways dirty/clean - Other (*specify*)   ________________  __ |
|  | 1. Training | - Vademecum/guidelines - Video-lessons - Courses (*face to face* and remote) - Simulation courses |
|  | 1. Digital Technology | - Implementation Wi-fi network - pc computers - tablets - smartphone |
|  | 1. Well-being | - Physical well being (*specify)*   *------------------------*   - Mental well being *(specify)*   ______________ |
| Patients | 1. Safety and positive/negative flow management | - Automated doors - Specific furnishings (e.g. stretchers, mattresses,..) - Specific buildings - Isolation system chambres - Isolation system stretchers - Other (specify)   ________________ |
|  | 1. Humanization of care | - Digital instruments (e.g. tablets, smartphones,…) - Prefabricated buildings - End of life area - Others (*specify*)   ________________ |

**Section 4.** *Intervention goals.* This section focused on the intervention goals implemented in order to optimize the response of each healthcare facility to COVID-19.

| **INTERVENTIONS GOALS** | | |
| --- | --- | --- |
| **SAFETY** | | |
| **Group** | **Intervention** | **Notes** |
| Healthcare workers | Wateer, Sanitation and Hygiene (WASH) points |  |
|  | Automated door |  |
|  | Lockers for changing room (double compartment) |  |
|  | Personal Protective Equipment (PPE):   - gloves - masks - gowns medical |  |
| Patients | - Automated door |  |
|  | - Spaces to be able to carry out interviews with future and actual patients |  |
|  | Purchase of:   - stretchers - mattresses - cushions - gazebo |  |
| Caregivers | - WASH points - Automated door - Gazebo |  |
